# Influence of protocol variables on outcomes of the star excursion balance test group (SEBT, mSEBT, YBT-LQ) in healthy individuals: a systematic review

**DOI:** 10.3389/fphys.2024.1415887

**Published:** 2024-08-02

**Authors:** Bartosz Zając, Maciej Olszewski, Anna Mika

**Affiliations:** ^1^ Laboratory of Functional Diagnostics, Central Scientific and Research Laboratory, University of Physical Education in Kraków, Kraków, Poland; ^2^ Doctoral School, University of Physical Education in Kraków, Kraków, Poland; ^3^ Institute of Clinical Rehabilitation, University of Physical Education in Kraków, Kraków, Poland

**Keywords:** star excursion balance test, y-balance test, postural stability, postural control, dynamic balance, limits of stability, test protocol, test results

## Abstract

**Background:**

The “SEBT group,” which includes the Star Excursion Balance Test (SEBT), its modified version (mSEBT), and the Lower Quarter Y-Balance Test (YBT-LQ), is used to assess the limits of stability. Interestingly, the testing protocol allows users a considerable degree of flexibility, which can affect the obtained results. Therefore, the objective of this systematic review was to analyze the impact of different protocol variants within the “SEBT group” on outcomes.

**Methods:**

Data were acquired by searching 4 databases (MEDLINE, ScienceDirect, Wiley, Springer Link) focusing on studies published in English in peer-reviewed journals, empirical in nature, conducted on healthy individuals, and examining the effects of various protocol variants on test outcomes. Study quality was assessed with the NHLBI quality assessment tool for pre-post studies with no control group.

**Results:**

The calculation method based on the maximum repetition yields statistically significantly higher results compared to other calculation methods. Allowing unrestricted arm movements during the test results in statistically significantly higher scores compared to the procedure that restricts arm movements. The impact of a warm-up, wearing footwear during testing, and using a dedicated kit remains ambiguous. To obtain reliable results, 4–6 familiarization trials are necessary, though fewer may suffice for athletes experienced in performing the test.

**Conclusion:**

This systematic review highlights the significant impact of the calculation method and arm movement restrictions on the outcomes of the “SEBT group.” The effects of wearing footwear during testing, warm-up, and using a dedicated test kit remain unclear. The required number of familiarization repetitions may varies depending on biological maturity level of the person being tested. Future research should develop a warm-up protocol tailored to the needs of the “SEBT group,” and investigate the impact of heel elevation during testing on outcomes.

**Systematic review registration:**

The protocol for this systematic review was prospectively registered in the OSF Registries (https://doi.org/10.17605/OSF.IO/JSKH2).

## 1 Introduction

Postural stability is the ability to actively maintain the vertical projection of body’s center of gravity within the support area (Andreeva et al., 2021). One dimension of postural stability is the limits of stability (LoS), which define the ranges of the body’s center of gravity shifts in various directions that do not lead to loss of balance (Melzer et al., 2008). A popular method of assessing LoS is “SEBT group,” which includes the Star Excursion Balance Test (SEBT), its modified version (mSEBT), and the Lower Quarter Y-Balance Test (YBT-LQ). A major advantage of these tests is their relatively low cost and user-friendliness, making them accessible not just for large sports and rehabilitation centers but also for smaller physiotherapy practices and sports clubs. The test results are primarily used for assessing the risk of injury (Gribble et al., 2012; Plisky et al., 2021), evaluating the outcomes of interventions (Chaabene et al., 2021), and are also considered as criteria for returning to sports (Oleksy et al., 2021). All these applications are extremely valuable from a training practice perspective, as they provide coaches and instructors with key insights into an athlete’s readiness and physical status.

The “SEBT group” directly measure the reach distance of the lower limbs (Kinzey and Armstrong, 1998; Plisky et al., 2009). The SEBT measures reach in 8 directions, whereas mSEBT and YBT-LQ are focused on 3 directions, utilizing a specialized test kit for the latter. The reduction in the number of directions in mSEBT and YBT-LQ stems from a desire to increase the test’s efficiency and to eliminate redundancies (Plisky et al., 2009). By focusing on 3 key directions - anterior (ANT), posterolateral (PL), and posteromedial (PM) – mSEBT and YBT-LQ offers a quicker and more focused assessment, which still effectively assess LoS but in a more practical manner, especially suitable for clinical environments. In each of the tests involves the participant standing on one leg and reaching as far as possible with the opposite lower limb in the designated directions. From these tests, several outcomes are obtained: (a) absolute (in cm) and normalized reach (in % lower limb length); (b) absolute and normalized composite score; and (c) interlimb ratio of the outcomes mentioned in points a and b.

A very important characteristic of each test is its validity, which informs whether the test measures what it was designed to measure, and its reliability, which indicates whether the test consistently measures what it is intended to measure. Research indicates that the “SEBT group” have been quite thoroughly examined from this perspective. Research conducted by Plisky et al. (2021) revealed significant differences in “SEBT group” performance among populations, thereby emphasizing the discriminative validity of these tools. Conversely, the relationship between the results of the “SEBT group” and the risk of future injuries (predictive validity) remains unclear. Many indications suggest that injury risk prediction based on “SEBT group” results is justified only for specific populations (Plisky et al., 2021) after applying standardized cutoff values (Lehr et al., 2013). Glave et al. (2016), comparing SEBT results with the LoS test using the Biodex Balance System, found a negative correlation. This suggests that the testing of postural stability through these methods is highly specific, as participants who performed well on one test were likely to score poorly on the other, indicating the unique and distinct nature of each test’s assessment of LoS. Furthermore, a systematic review conducted by Powden et al. (2019) demonstrated excellent inter- and intra-rater reliability of YBT-LQ results in healthy adults, a crucial aspect indicating that the test outcomes are repeatable and consistent regardless of the evaluator (inter-rater reliability) or the timing of the assessment (intra-rater reliability), which is a necessary condition for utilizing this tool in clinical decision-making.

Interestingly, the testing procedure of the “SEBT group” allows users a considerable degree of flexibility, which can affect the results. This flexibility pertains to the choice of calculation method, restrictions on arm movements, wearing footwear during testing, warm-up, the number of familiarization repetitions, the use of a dedicated test kit, and restrictions on heel lifting. With this in mind, a review of studies analyzing the impact of these protocol variables can serve as a useful source of information for selecting the most optimal combination of variables for a given issue. Additionally, the review will provide data that can be used to estimate adjustments when comparing results obtained using different protocols. Therefore, this systematic review aimed to analyze the impact of different protocol variants within the “SEBT group” on outcomes. To the best of the authors’ knowledge, this is the first study to address this issue. Its completion will result in the creation of a valuable source of information that will be useful from both a research and clinical practice perspective.

## 2 Methods

### 2.1 Protocol and registration

The protocol for this systematic review was prospectively registered in the OSF Registries (https://doi.org/10.17605/OSF.IO/JSKH2). The systematic review was conducted in accordance with the Preferred Reporting Items for Systematic Reviews and Meta-Analyses guidelines (Page et al., 2021).

### 2.2 Search strategy and study selection

The systematic review was conducted in February 2024 by searching through 4 databases: MEDLINE (using PubMed search engine), ScienceDirect, Wiley and Springer Link, specifically targeting studies published after the year 1998 - the year of publication of the first work using SEBT (Kinzey and Armstrong, 1998). The search strategy, as detailed in Table 1, encompassed a wide range of terms related to YBT-LQ and SEBT, along with related procedural aspects. B.Z and M.O independently reviewed the titles and abstracts of the identified studies during the search, then analyzed the full texts of relevant studies, and finally compiled a list of qualified research. Next, both lists were compared and discussed. In case consensus could not be reached, the final decision was made based on the opinion of 3rd author A.M. In managing the references, Mendeley Reference Manager was employed, facilitating efficient organization to the literature sources.

**TABLE 1:** Search strategy.

Database	Search command
MEDLINE	("Y-Balance Test"[tiab] OR YBT[tiab] OR "Star Excursion Balance Test"[tiab] OR "Star Excursion Test" OR SEBT[tiab]) AND ( "warm-up exercise"[MeSH] OR "warm-up"[tiab] OR ((attempt*[tiab] OR trial*[tiab] OR repetition*[tiab]) AND number*[tiab]) OR ((maximum[tiab] OR average[tiab] OR mean[tiab]) AND reach[tiab]) OR shoes[MeSH] OR shoe*[tiab] OR footwear[tiab] OR barefoot[tiab] OR insole[tiab] OR hand*[tiab] OR arm[tiab] OR "upper limb"[tiab] OR heel[tiab] OR procedure[tiab] OR guideline*[tiab] OR manual[tiab] OR standard*[tiab] OR ((kit[tiab] OR set[tiab] OR suit[tiab]) AND test*[tiab]) )
ScienceDirect	Search 1 (“Y-Balance Test” OR YBT OR “Star Excursion Balance Test” OR SEBT OR “Star Excursion Test”) AND warm-up
	Search 2 ("Y-Balance Test” OR YBT OR “Star Excursion Balance Test” OR SEBT OR “Star Excursion Test”) AND ((attempt OR trail OR repetition) AND number)
	Search 3 ("Y-Balance Test” OR YBT OR “Star Excursion Balance Test” OR SEBT OR “Star Excursion Test”) AND ((maximum OR average OR mean) AND reach)
	Search 4 ("Y-Balance Test” OR YBT OR “Star Excursion Balance Test” OR SEBT OR “Star Excursion Test”) AND (shoe OR footwear OR barefoot OR sole)
	Search 5 ("Y-Balance Test” OR YBT OR “Star Excursion Balance Test” OR SEBT″OR “Star Excursion Test”) AND (hand OR arm OR upper limb OR heel)
	Search 6 ("Y-Balance Test” OR YBT OR “Star Excursion Balance Test” OR SEBT OR “Star Excursion Test”) AND (procedure OR guideline OR manual OR standard)
	Search 7 ("Y-Balance Test” OR YBT OR “Star Excursion Balance Test” OR SEBT OR “Star Excursion Test”) AND ((kit OR set OR suit) AND test)
Wiley	("Y-Balance Test" OR YBT OR "Star Excursion Balance Test" OR "Star Excursion Test" OR SEBT) AND ( "warm-up" OR ((attempt* OR trial* OR repetition*) AND number*) OR ((maximum OR average OR mean) AND reach) OR shoes OR shoe* OR footwear OR barefoot OR insole OR hand* OR arm OR "upper limb" OR heel OR procedure OR guidelin* OR manual OR standard* OR ((kit OR set OR suit) AND test*) )
	Comment: The above code was used for searching in titles, abstracts, and keywords.
Springer Link	("Y-Balance Test" OR YBT OR "Star Excursion Balance Test" OR "Star Excursion Test" OR SEBT) AND ( "warm-up" OR ((attempts OR trials OR repetitions) AND number) OR ((maximum OR average OR mean) AND reach) OR shoes OR shoe OR footwear OR barefoot OR insole OR hands OR arms OR "upper limb" OR heel OR procedure OR guideline OR manual OR standard OR ((kit OR set OR suit) AND test) )
	Comment: The following filters were used: (1) content type: articles; (2) date published 1998–2024; (3) languages: English; (4) disciplines: medicine and public health, life sciences, biomedicine

### 2.3 Eligibility criteria

For this systematic review, the following eligibility criteria were applied: (i) publication in English (full text) in a peer-reviewed journal; (ii) cross sectional and experimental study design (review articles, editorials, speeches, comments, abstracts, case studies, and surgical procedures were not considered); (iii) comprising individuals of all ages who are healthy, with no history of major lower limb injuries or surgeries, and no diagnosed issues with postural control. In terms of the intervention (iv), the studies may explore the following aspects of the “SEBT group” protocol: choice of calculation method based on the maximum repetition, conducting test with restricted arm movements, performing the test in footwear, conducting a warm-up before testing, preceding test repetitions with 6 familiarization repetitions, using a dedicated test kit, and allowing heel lifting during testing. Regarding the comparator (v), it might include: choice of calculation method not based on the maximum repetition, conducting test without restricted arm movements, performing the test barefoot, not conducting a warm-up before testing, preceding test repetitions with a number of familiarization repetitions other than 6, not using of a dedicated test kit, and not allowing heel lifting during testing Finally, the outcome (vi) will focus on both absolute and normalized reaches, as well as a composite score.

### 2.4 Methodological quality assessment

The quality of the included studies was assessed using the NHLBI quality assessment tool for before-after (pre-post) studies without a control group (NHLBI, 2022). This evaluation was conducted independently by B.Z. and M.O. It involved selecting one of 5 options for each of 12 items: “yes,” “no,” “cannot determine/unclear,” “not reported,” or “not applicable.” The total score was calculated as the sum of “yes” responses divided by the number of eligible items, expressed as a percentage. Items marked as “not applicable” were not taken into account when calculating the total score. Subsequently, the overall rating was categorized into one of 3 groups based on the total score: poor (<25%), fair (25%–75%), or good (>75%). After the independent assessments, the results were compared and discussed. In cases where consensus was not reached, the opinion of a 3rd author, A.M., was sought.

### 2.5 Data extraction, grouping and analysis

Using a standardized form, researchers B.Z. and M.O. independently extracted specific data from each study, focusing on descriptors such as sample size, age, gender, and health conditions. Additionally, outcomes obtained using different protocols or the differences between them were collected, employing measures of central tendency (mean) and dispersion (standard deviation or range). The probability of type I error and/or the effect size (η_p_^2^, Cohen’s d) were also determined. Moreover, reliability measures, including the intraclass correlation coefficient (ICC), standard error of measurement (SEM), minimal detectable change (MDC), or smallest detectable difference (SDD) were extracted. The data were systematically compared and discussed. In cases where consensus was not reached, a 3rd researcher, A.M., made the final decision. The extracted data were then categorized based on the variables of the “SEBT group” protocols to understand the impact of each variable on the test outcomes. Subsequently, the data were analyzed in a narrative format.

## 3 Results

**FIGURE 1:**
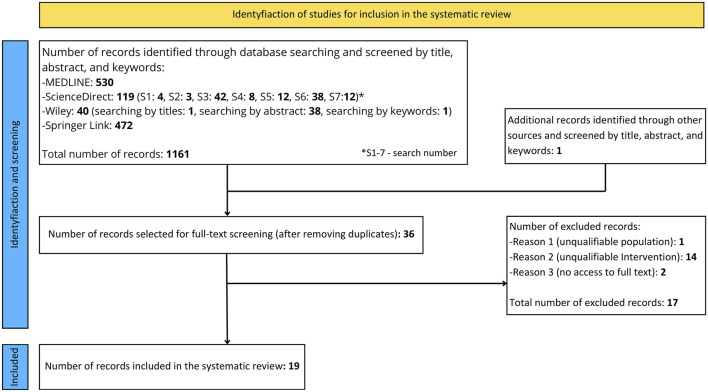
Systematic review flowchart.

In the systematic review, 19 studies were ultimately included. A summary of the database search and selection process is depicted in Figure 1. The methodological quality of the 6 studies included in the systematic review was assessed as “good,” while the remaining 13 were assessed as “fair.” A detailed assessment of the studies can be found in Table 2. In Table 3, a summary of the methods for standardizing test protocols in the studies included in the review is presented.

**TABLE 2:** Methodological quality of included studies.

Author	Item 1	Item 2	Item 3	Item 4	Item 5	Item 6	Item 7	Item 8	Item 9	Item 10	Item 11	Item 12	Total score [%]	Quality rating
Shaffer et al. (2013)	●●●	●●●	●●●	NR	●●	●●●	●●●	●●●	NA	●●●	NA	NA	77.8	Good
Sokulska et al. (2024)	●●●	●●●	●●●	NR	●●●	●●●	●●●	NR	NA	●●●	NA	NA	77.8	Good
Objero et al. (2019)	●●●	●	●●	NR	●●●	●●●	●●●	NR	NA	●●●	NA	NA	55.6	Fair
Hébert-Losier (2017)	●●●	●●	●●●	NR	●●	●●●	●●●	NR	NA	●●●	NA	NA	55.6	Fair
Muehlbauer et al. (2022b)	●●●	●	●●●	NR	●●	●	●●●	NR	NA	●●●	NA	NA	44.4	Fair
Muehlbauer et al. (2022a)	●●●	●	●●●	NR	●●●	●●	●●●	NR	NA	●●●	NA	NA	55.6	Fair
Sogut et al. (2022)	●●●	●●●	●●●	NR	●●●	●●●	●●●	NR	NA	●●●	NA	NA	77.8	Good
Park et al. (2023)	●●	●●●	●●	NR	●●	●●●	●●●	●	NA	●●●	NA	NA	44.4	Fair
Bizzini et al. (2013)	●●●	●	●●	NR	●●	●	●●●	NR	NA	●●●	●●●	NA	40.0	Fair
Gogte et al. (2017)	●●●	●●●	●●	●●●	●●●	●	●●	NR	●●●	●●●	●●●	NA	63.6	Fair
Imai et al. (2014)	●●●	●●●	●	NR	●	●●●	●●●	NR	NR	●●●	●●●	NA	54.5	Fair
Belkhiria-Turki et al. (2014)	●●●	●●●	●●●	NR	●●	●●●	●●●	NR	NR	●●●	●●●	NA	63.6	Fair
Linek et al. (2017)	●●●	●●●	●●	NR	●●	●●●	●●●	NR	NA	●●●	NA	NA	55.6	Fair
Kattilakoski et al. (2023)	●●●	●●●	●	NR	●	●●●	●●●	NR	NA	●●●	NA	NA	55.6	Fair
Onofrei et al. (2019)	●●●	●●●	●●●	NR	●●●	●●●	●●●	NR	NA	●●●	NA	NA	77.8	Good
Munro and Herrington (2010)	●●●	●●●	●●	NR	●●	●●●	●●●	NR	NR	●●●	●●●	NA	54.5	Fair
Robinson and Gribble (2008)	●●●	●	●●	NR	●●	●●●	●●●	NR	NA	●●●	NA	NA	44.4	Fair
Bulow et al. (2019)	●●●	●●●	●●●	NR	●●●	●●●	●●●	NR	NA	●●●	NA	NA	77.8	Good
Jagger et al. (2020)	●●●	●●●	●●●	NR	●●●	●●●	●●●	NR	NA	●●●	NA	NA	77.8	Good
Total score [%], median (1st and 3rd quartile)													**55.6 (54.5–77.8)**	

Item 1. Was the study question or objective clearly stated?

Item 2. Were eligibility/selection criteria for the study population prespecified and clearly described?

Item 3. Were the participants in the study representative of those who would be eligible for the test/service/intervention in the general or clinical population of interest?

Item 4. Were all eligible participants that met the prespecified entry criteria enrolled?

Item 5. Was the sample size sufficiently large to provide confidence in the findings?

Item 6. Was the test/service/intervention clearly described and delivered consistently across the study population?

Item 7. Were the outcome measures prespecified, clearly defined, valid, reliable, and assessed consistently across all study participants?

Item 8. Were the people assessing the outcomes blinded to the participants' exposures/interventions?

Item 9. Was the loss to follow-up after baseline 20% or less? Were those lost to follow-up accounted for in the analysis?

Item 10. Did the statistical methods examine changes in outcome measures from before to after the intervention? Were statistical tests done that provided p values for the pre-to-post changes?

Item 11. Were outcome measures of interest taken multiple times before the intervention and multiple times after the intervention (i.e., did they use an interrupted time-series design)?

Item 12. If the intervention was conducted at a group level (e.g., a whole hospital, a community, etc.) did the statistical analysis take into account the use of individual-level data to determine effects at the group level?

Legend: ●●●, yes. ●●, unclear/cannot determine. ●, no.

NR, not reported; NA, not applicable. The bold values are the median (1st and 3rd quartile).

**TABLE 3:** Method of standardizing test protocols in included studies.

Author	Item 1	Item 2	Item 3	Item 4	Item 5	Item 6	Item 7
Shaffer et al. (2013)	●	●●● (6)	●●●	●●●	●	●●●	●●●
Sokulska et al. (2024)	●	●●● (6)	●	●●●	●●●	●	●●●
Objero et al. (2019)	●	●●● (3)	P	●●●	●●	●●●	●●●
Hébert-Losier (2017)	●●●	●●● (6)	P	●●●	●	●●●	●●●
Muehlbauer et al. (2022b)	●	●●● (3)	P	●●	●●	●	●
Muehlbauer et al. (2022a)	●	●●● (3)	P	●●●	●	●	●●●
Sogut et al. (2022)	●●●	●●● (4)	P	P	●	●	●●●
Park et al. (2023)	●	●●● (untimed)	●●●	P	●	●●●	●●●
Bizzini et al. (2013)	P	●	●●	●●	●	●●	●●●
Gogte et al. (2017)	P	●	●●	●●	●●	●	●
Imai et al. (2014)	P	●●● (6)	●●●	●●●	●	●●●	●●●
Belkhiria-Turki et al. (2014)	P	●●● (6)	●●●	●●●	●	●●●	●●●
Linek et al. (2017)	●	P	●●●	●●●	●●	●●●	●●●
Kattilakoski et al. (2023)	●●●	●●● (1)	●●●	●●●	●	●●●	●●●
Onofrei et al. (2019)	●	●●● (1)	●●●	●●●	●	●●●	●●●
Munro and Herrington (2010)	●	P	●●●	●●●	●	●●●	●●●
Robinson and Gribble (2008)	●	P	●●●	●●●	●	●●●	●●●
Bulow et al. (2019)	●	●●● (4)	●●●	●●●	●	●●●	●●●
Jagger et al. (2020)	●	●●● (3)	●●●	●●●	●	●●●	●●●

Item 1. Was a warm-up conducted before the test?

Item 2. Were familiarization trials performed before the test? If so, what was their number?

Item 3. Were arm movements restricted?

Item 4. Was the test conducted barefoot?

Item 5. Was the heel lift allowed?

Item 6. Was the order of trials in each direction specified?

Item 7. Were errors that resulted in a trial being disqualified specified?

Legend: ●●●, yes. ●●, unclear. ●, no.

P, purpose of the study to examine this variable under different conditions

### 3.1 Choice of calculation method

A study conducted by Sokulska et al. (2024) demonstrated that the method of calculating scores based on the maximum repetition, compared to the average of 3 repetitions, yields statistically significantly higher normalized scores in each direction and a higher composite score (the differences range between 1.8% and 2.8%). Conversely, the study by Shaffer et al. (2013) indicates that the method of calculating scores based on the average of 3 repetitions, compared to the method based on the maximum repetition, is characterized by more favorable reliability indicators, i.e., higher ICC as well as lower SEM and MDC. In contrast to these findings, the study by Kattilakoski et al. (2023) which compared methods based on the first 3 repetitions, the best 3 repetitions, and the maximum repetition, showed that the method of calculating results does not affect reliability indicators. Detailed data can be found in Table 4.

**TABLE 4:** Summary of study results on the impact of the choice of calculation method on test outcomes.

Test	Authors	Participants	Difference Magnitude/Stat. Significance/Effect size						Reliability indicators					
YBT-LQ	Shaffer et al. (2013)	64 service members (♀11, ♂53), mean ± SD age: 25.2 ± 3.8			LL		RL		ICC (95%CI)		SEM (cm)		MDC (cm)	
			ANT (%LLL), mean ± SD	max	66.0 ± 7.8		65.8 ± 7.6		0.82 (0.72–0.89		3.1		8.7	
				avg	63.6 ± 7.2		63. ±7.7		0.93 (0.88–0.96)		2.0		5.5	
			PM (%LLL), mean ± SD	max	105.3 ± 8.3		104.6 ± 8.9		0.81 (0.71–0.88)		3.7		10.3	
				avg	102.7 ± 8.6		102.0 ± 9.4		0.91 (0.85–0.94)		2.7		7.5	
			PL (%LLL), mean ± SD	max	100.5 ± 9.1		101.4 ± 9.6		0.80 (0.68–0.87)		4.2		11.5	
				avg	97.2 ± 9.4		98.2 ± 10.0		0.85 (0.76–0.91)		3.5		9.7	
			CS (%LLL), mean ± SD	max	90.6 ± 7.5		90.6 ± 7.9 87.9 ± 8.3		0.85 (0.76–0.91)		9.0		24.8	
				avg	87.8 ± 7.6				0.91 (0.85–0.95)		7.0		19.5	
YBT-LQ	Sokulska et al. (2024)	100 healthy individuals ♀ 48, mean ± SD age: 23.4 ± 2.1 ♂ 52, mean ± SD age: 23.4 ± 2.1			KL		SL		Not applicable					
			ANT (% DIFF), mean ± SD (*p*-value)		1.8 ± 1.2 (<0.001*)		2.0 ± 1.7 (<0.001*)							
			PM (% DIFF), mean ± SD (*p*-value)		2.7 ± 1.8 (<0.001*)		2.2 ± 2.4 (<0.001*)							
			PL (% DIFF), mean ± SD (*p*-value)		2.8 ± 1.9 (<0.001*)		2.2 ± 1.3 (<0.001*)							
			CS (% DIFF), mean ± SD (*p*-value)		2.4 ± 1.1 (<0.001*)		2.1 ± 0.9 (<0.001*)							
mSEBT	Kattilakoski et al. (2023)	16 healthy individuals ♀12, mean ± SD age: 37.9 ± 6.9 ♂4, mean ± SD age: 42.5 ± 5.7			F3	B3		max		F3	B3	max		
			ANT (%LLL), mean ± SD		67.5 ± 4.7	69.1 ± 4.6		69.9 ± 4.5	ICC (95%CI) SEM% MDC	0.86 (0.68–0.94) 4.11 7.6	0.90 (0.78–0.95) 3.28 6.2	0.89 (0.74–0.95) 3.46 6.7		
			PM (%LLL), mean ± SD		97.8 ± 9.7	101.5 ± 9.5		102.4 ± 9.6	ICC (95%CI) SEM MDC	0.82 (0.63–0.91) 5.70 15.6	0.83 (0.65–0.92) 5.14 14.6	0.83 (0.74–0.95) 5.11 14.6		
			PL (%LLL), mean ± SD		95.5 ± 9.2	98.9 ± 8.7		100.1 ± 8.5	ICC (95%CI) SEM MDC	0.84 (0.67–0.92) 5.49 14.5	0.89 (0.78–0.95) 4.12 11.3	0.89 (0.78–0.95) 3.99 11.1		

%LLL, percentage of lower limb length; ANT/PM/PL and CS, anterior/posterolateral/posteromedial reach and composite score; LL/RL, left/right leg; KL/SL, kicking/stance leg; max/avg, calculation method based on maximum repetition/average of 3 repetitions, F3/B3, calculation method based on first 3 repetitions/best 3 repetition; %DIFF, percentage difference between max and avg; ICC (95%CI), interclass correlation coefficient with 95% confidence interval; SEM, standard error of measurement percentage; MDC, minimal detectable change; *p*-value, probability of type I error, SD – standard deviation.

### 3.2 Arm movement restriction

Most studies indicate that the test procedure without arm movement restrictions, compared to the procedure with restrictions, yields statistically significantly higher scores in each direction and a higher composite score, regardless of the age and gender of the test subject, as well as the use of footwear during the test (Hébert-Losier, 2017; Objero et al., 2019; Muehlbauer et al., 2022b; 2022a; Sogut et al., 2022). Conversely, the study by Sogut et al. (2022) indicates that the test procedure with arm movement restrictions, compared to the procedure without restrictions, is characterized by better reliability indicators, i.e., higher ICC values and lower SEM and MDC values. Detailed data can be found in Table 5.

**TABLE 5:** Summary of study results on the impact of arm movement restrictions on test outcomes.

Test	Authors	Participants	Difference Magnitude/Stat. Significance/Effect size									Reliability indicators	
YBT-LQ	Objero et al. (2019)	20 healthy individuals (♀10, ♂10) mean ± SD age: 20.7 ± 1.3			LL (RA vs. NRA)				RL (RA vs. NRA)			Not applicable	
			ANT, *p*-value		<0.05*				<0.05*				
			PM, *p*-value		<0.05*				<0.05*				
			PL, *p*-value		<0.05*				<0.05*				
			CS, *p*-value		<0.05*				<0.05*				
YBT-LQ	Hébert-Losier (2017)	46 healthy individuals ♀23, mean ± SD age: 23.5 ± 2.5 ♂23, mean ± SD age: 25.7 ± 4.6			RA				NRA		*p*-value	Not applicable	
			ANT (%LLL), mean		75.1				76.0		<0.05*		
			PM (%LLL), mean		119.0				123.2		<0.05*		
			PL (%LLL), mean		117.1				121.9		<0.05*		
			CS (%LLL), mean		103.7				107.0		<0.05*		
YBT-LQ	Muehlbauer et al. (2022b)	40 healthy children ♀22, mean ± SD age: 11.5 ± 0.6 ♂18, mean ± SD age: 11.5 ± 0.6					RA		NRA		*p*-value (η_p_^2^)	Not applicable	
			ANT (%LLL), mean		B		77.3 ± 7.8		79.3 ± 7.8		<0.001* (0.36)		
					G		73.0 ± 9.7		77.1 ± 9.1				
			PM (%LLL), mean ± SD		B		108.1 ± 12.1		113.1 ± 11.9		<0.001* (0.38)		
					G		102.9 ± 13.6		109.0 ± 16.6				
			PL (%LLL), mean ± SD		B		103.7 ± 13.6		108.3 ± 13.5		<0.001* (0.26)		
					G		101.0 ± 13.1		105.8 ± 15.4				
			CS(%LLL), mean ± SD		B		96.4 ± 10.3		100.2 ± 9.9		<0.001* (0.53)		
					G		92.3 ± 11.1		97.3 ± 12.6				
YBT-LQ	Muehlbauer et al. (2022a)	111 healthy individuals 40 children, mean ± SD age: 11.5 ± 0.6 30 adolescent, mean ± SD age 14.0 ± 1.1 41 young adults, mean ± SD: 24.7 ± 3.0							RA vs. NRA			Not applicable	
			ANT, *p*-value (Cohen’s d)						<0.001* (0.32)				
			PM, *p*-value (Cohen’s d)						<0.001* (0.52)				
			PL, *p*-value (Cohen’s d)						<0.001* (0.47)				
			CS, *p*-value (Cohen’s d)						<0.001* (0.65)				
mSEBT	Sogut et al. (2022)	51 healthy individuals (♀21, ♂30) mean age±SD: 22.7 ± 1.9				RA		NRA		*p*-value		ICC, SEM(%), MDC (%)	
												RA	NRA
			ANT (%LLL), mean	WS		76.6		76.2		≥0.05		0.98, 1.05, 2.39	0.94, 2.14, 3.41
				B		74.6		74.8		≥0.05		0.97, 1,12, 2,47	0.95, 1.72, 3.06
			PM (%LLL), mean	WS		99.8		104.6		<0.05*		0.98, 1.35, 2,71	0.98, 1.40, 2.76
				B		96.8		102.3		<0.05*		0.98, 1.35, 2.71	0.98, 1.32, 2.68
			PL (%LLL), mean	WS		93.4		98.2		<0.05*		0.99, 1.30, 2.66	0.97, 2.0, 3.31
				B		91.8		97.4		<0.05*		0.98, 1.24, 2.60	0.98, 1.33, 2.69
			CS (%LLL), mean	WS		89.9		93.1		<0.05*		0.99, 0.79, 2.08	0.98, 1.09, 2.43
				B		87.7		91.5		<0.05*		0.99, 0.83, 2.12	0.98, 0.94, 2.27

%LLL, percentage of lower limb length; ANT/PM/PL and CS, anterior/posterolateral/posteromedial reach and composite score; LL/RL, left/right leg; B/G, boys/girls; RA, restricted arm movement; NRA, non-restricted arm movement; WS/B, with shoes/barefoot; ICC (95%CI), interclass correlation coefficient with 95% confidence interval; SEM, standard error of measurement percentage; MDC, minimal detectable change; *p*-value, probability of type I error; SD, standard deviation.

### 3.3 Wearing footwear during testing

The results of the study by Sogut et al. (2022) indicate that performing the test in footwear, compared to testing without footwear, yields statistically significantly higher score in the PM direction and composite score (regardless of whether the trial was performed with arm movement restrictions), as well as in the ANT direction (in the case of the procedure with arm movement restrictions). Conversely, the results of the study by Park et al. (2023) indicate that performing the test in footwear with regular insoles, compared to performing the test barefoot, yields statistically significantly higher scores in the PL direction for the dominant leg. Additionally, performing the test in footwear with textured insoles, compared to performing the test barefoot, yields statistically significantly higher scores in the PM and PL directions for both legs. Detailed data can be found in Table 6.

**TABLE 6:** Summary of study results on the impact of wearing footwear during testing on test outcomes.

Test	Authors	Participants	Difference Magnitude/Stat. Significance/Effect size									Reliability indicators	
mSEBT	Sogut et al. (2022)	51 healthy individuals (♀21, ♂30) mean age±SD: 22.7 ± 1.9					WS		B		*p*-value	ICC, SEM(%), MDC (%)	
												WS	B
			ANT (%LLL), mean		RA		76.6		74.6		<0.05*	0.98, 1.05, 2.39	0.97, 1.12, 2.47
					NRA		76.2		74.8		≥0.05	0.94, 2.14, 3.41	0.95, 1.72, 3.06
			PM (%LLL), mean		RA		99.8		96.8		<0.05*	0.98, 1.35, 2,71	0.98, 1.35, 2.71
					NRA		104.6		102.3		<0.05*	0.98, 1.40, 2.76	0.98, 1.32, 2.68
			PL (%LLL), mean		RA		93.4		91.8		≥0.05	0.99, 1.30, 2.66	0.98, 1.24, 2.60
					NRA		98.2		97.4		≥0.05	0.97, 2.0, 3.31	0.98, 1.33, 2.69
			CS (%LLL), mean		RA		89.9		87.7		<0.05*	0.99, 0.79, 2.08	0.99, 0.83, 2.12
					NRA		93.1		91.5		<0.05*	0.98, 1.09, 2.43	0.98, 0.94, 2.27
YBT-LQ	Park et al. (2023)	20 healthy individuals (♀12, ♂8) mean (range) age: 23 (18–29)					SRI vs. B		STI vs. B		MS vs. B	Not applicable	
			ANT, *p*-value		D		≥0.05		≥0.05		≥0.05		
					ND		≥0.05		≥0.05		≥0.05		
			PM, *p*-value		D		≥0.05		<0.05*		≥0.05		
					ND		≥0.05		<0.05*		≥0.05		
			PL, *p*-value		D		<0.05*		<0.05*		≥0.05		
					ND		≥0.05		<0.05*		≥0.05		

%LLL, percentage of lower limb length; ANT/PM/PL and CS, anterior/posterolateral/posteromedial reach and composite score; WS/B, with shoes/barefoot; SRI/STI, shoes with regular/texture insoles; MS, minimalist shoes; ICC, interclass correlation coefficient; SEM, standard error of measurement; MDC, minimal detectable change; *p*-value, probability of type I error; SD, standard deviation

### 3.4 Warm-up

The results of the study by Bizzini et al. (2013) indicate that preceding the test with the “FIFA 11+” warm-up significantly increases the composite score compared to testing without a warm-up. Similarly, the study by Imai et al. (2014) shows that a warm-up including trunk stabilization exercises significantly increases the scores in the PM and PL directions, as well as the composite score, compared to testing without a warm-up. Conversely, the study conducted by Gogte et al. (2017) did not show statistically significant differences in the composite score for tests preceded by active, passive, and combined warm-ups. Additionally, the results of the study conducted by Belkhiria-Turki et al. (2014) mainly indicate an unclear, trivial, or small effect size of including static or dynamic stretching exercises in the warm-up preceding the test, regardless of the number of repetitions. Detailed data can be found in Table 7.

**TABLE 7:** Summary of study results on the impact of warm-up on test outcomes.

Test	Authors	Participants	Difference Magnitude/Stat. Significance/Effect size											Reliability indicators
SEBT	Bizzini et al. (2013)	20 amateur ♂ football players mean ± SD age: 25.5 ± 5.1				Baseline	After FIFA 11+ WU			Change (%), mean (95%CI)		*p*-value		Not applicable
			CS (%LLL), mean ± SD			84.6 ± 6.9	86.4 ± 6.8			2.9 (1.9–3.9)		<0.001*		
mSEBT	Imai et al. (2014)	11♂ adolescent soccer players mean ± SD age: 17.9 ± 0.3				Baseline	After WU			Change (%), mean		*p*-value (Cohen’s d)		ICC
			ANT (%LLL), mean ± SD	SE		74.0 ± 3.4	73.7 ± 4.6			0.4		≥0.05 (0.07)		0.965
				CE		75.0 ± 6.0	74.9 ± 5.1			0.1		≥0.05 (0.01)		
				NE		73.3 ± 5.3	73.5 ± 4.8			0.3		≥0.05 (0.01)		
			PM (%LLL), mean ± SD	SE		105.3 ± 5.8	109.8 ± 6.4			4.3		<0.05*(0.74)		0.948
				CE		105.5 ± 7.6	106.2 ± 8.2			0.6		≥0.05 (0.08)		
				NE		106.6 ± 4.9	108.0 ± 4.4			1.3		≥0.05 (0.29)		
			PL (%LLL), mean ± SD	SE		102.8 ± 7.3	106.2 ± 8.1			3.3		<0.05*(0.44)		0.888
				CE		103.6 ± 6.8	105.2 ± 8.1			1.5		≥0.05 (0.21)		
				NE		105.4 ± 7.4	104.1 ± 7.8			−1.3		≥0.05 (0.17)		
			CS (%LLL), mean ± SD	SE		94.0 ± 4.8	96.8 ± 5.7			2.9		<0.05*(0.53)		Not applicable
				CE		94.7 ± 6.1	95.6 ± 6.5			1.0		≥0.05 (0.15)		
				NE		95.1 ± 5.1	95.4 ± 5.1			0.3		≥0.05 (0.06)		
SEBT	Gogte et al. (2017)	19 (♀♂) recreation sports players mean (SD) age: 21.1 ± 2.0						passive WU vs. active WU	active WU vs. combined WU		combined WU vs. passive WU			Not applicable
			CS (%LLL), mean ± SD (*p*-value)					0.262 (1.000)	−0.002 (1.000)		−0.260 (1.000)			
mSEBT	Belkhiria-Turki et al. (2014)	28 healthy individuals ♀13, mean ± SD age: 22.1 ± 0.3 ♂15, mean ± SD age: 22.7 ± 1.9						Baseline	After WU		Change (%), mean ± 95%CI		Effect size	Not applicable
			ANT (%LLL), mean ± SD		SWU4			88.3 ± 6.7	88.6 ± 6.2		0.42 ± 1.09		Trivial	
					SWU8			86.6 ± 5.3	87.4 ± 6.2		0.59 ± 1.54		Trivial	
					SWU12			86.5 ± 6.8	88.5 ± 7.6		2.26 ± 1.80		Small	
					DWU4			90.1 ± 7.8	91.9 ± 8.0		1.93 ± 1.07		Unclear	
					DWU8			89.3 ± 8.2	91.2 ± 8.9		2.07 ± 1.23		Unclear	
					DWU12			88.7 ± 7.4	91.5 ± 8.7		3.13 ± 1.92		Small	
			PM (%LLL), mean ± SD		SWU4			100.1 ± 6.9	102.5 ± 7.2		2.39 ± 1.24		Small	
					SWU8			99.7 ± 7.2	101.7 ± 6.9		2.05 ± 1.80		Small	
					SWU12			99.7 ± 7.4	103.0 ± 7.2		3.24 ± 1.94		Small	
					DWU4			103.4 ± 7.6	105.7 ± 6.9		2.20 ± 1.02		Small	
					DWU8			103.1 ± 7.1	105.8 ± 6.8		1.63 ± 1.19		Unclear	
					DWU12			102.9 ± 6.3	103.7 ± 7.6		0.76 ± 2.32		Unclear	
			PL (%LLL), mean ± SD		SWU4			96.8 ± 7.1	99.6 ± 7.7		2.83 ± 1.63		Small	
					SWU8			95.1 ± 7.1	97.9 ± 7.8		2.89 ± 1.36		Small	
					SWU12			97.3 ± 6.8	99.9 ± 7.5		2.63 ± 2.21		Small	
					DWU4			100.2 ± 5.9	103.1 ± 7.7		2.90 ± 1.40		Moderate	
					DWU8			100.8 ± 6.4	102.4 ± 7.1		1.52 ± 1.60		Unclear	
					DWU12			100.2 ± 6.3	102.1 ± 6.8		1.89 ± 1.61		Small	

%LLL, percentage of lower limb length, ANT/PM/PL and CS, anterior/posterolateral/posteromedial reach and composite score; SE/CE, warm-up with stabilization/convention trunk exercise; NE, non-exercise; WU, warm-up; S-WU4/8/12, static stretching within warm-up (volume 4/8/12 sets), D-WU4/8/12, dynamic stretching within warm-up (volume 4/8/12 sets); ICC, interclass correlation coefficient; *p*-value, probability of type I error, SD, standard deviation.

### 3.5 Number of familiarization repetitions

The results of studies conducted by Linek et al. (2017) and Kattilakoski et al. (2023) indicate that achieving a plateau in reach distances requires 6 familiarization repetitions. A test preceded by 6 familiarization repetitions is characterized by the following reliability indicators: ICC = 0.57–0.82, SEM = 3.30–5.90, and MDC = 7.68–13.5. Conversely, the studies by Munro and Herrington (2010), as well as Robinson and Gribble (2008), suggest that a plateau can be reached after 4 familiarization repetitions. A test preceded by 4 familiarization repetitions is characterized by the following reliability indicators: ICC = 0.84–0.92, SEM = 2.21–2.94, and SDD = 6.13–8.15. Additionally, the study conducted by Onofrei et al. (2019) indicates that for athletes with experience in performing the test, 1 familiarization repetition is sufficient to achieve consistent results. In the situation where the test is preceded by 1 familiarization repetition, the reliability indicators are: ICC = 0.90–0.94, SEM = 0.91–2.86, and MDC = 2.54–7.94. Detailed data can be found in Table 8.

**TABLE 8:** Summary of study results on the impact of number of familiarization repetitions on test outcomes.

Test	Authors	Participants	Number of familiarization repetitions – authors’ conclusion	Reliability indicators						
YBT-LQ	Linek et al. (2017)	38 adolescent footballers mean (range) age: 15.6 (14–17)	6 familiarization repetitions are required to reach a plateau	Reliability indicators between 7th, 8th, and 9th repetitions						
							ICC		SEM (%)	MDC (%)
				ANT	RL		0.66		3.67	8.54
					LL		0.68		3.30	7.68
				PM	RL		0.57		5.90	13.7
					LL		0.64		5.64	13.1
				PL	RL		0.70		5.81	13.5
					LL		0.82		4.41	10.3
mSEBT	Kattilakoski et al. (2023)	16 healthy individuals ♀12, mean ± SD age: 37.9 ± 6.9 ♂4, mean ± SD age: 42.5 ± 5.	6 familiarization repetitions are required to reach a plateau	Not applicable						
mSEBT	Onofrei et al. (2019)	122 healthy elite athletes (♀34, ♂88) mean ± SD age: 25.1 ± 5.1	1 familiarization repetitions is sufficient to achieve reliable resultsin healthy athletes already familiar with the test from previous assessments.	Reliability indicators between 2nd, 3rd, and 4th repetitions						
							ICC		SEM (%)	MDC (%)
				ANT		RL	0.90		0.91	2.54
						LL	0.93		1.66	4.61
				PM		RL	0.93		2.60	7.21
						LL	0.94		2.61	7.26
				PL		RL	0.94		2.53	7.02
						LL	0.93		2.86	7.94
				CS		RL	0.93		1.72	4.78
						LL	0.95		1.67	4.64
SEBT	Munro and Herrington (2010)	22 healthy individuals ♀11, mean ± SD age: 22.3 ± 3.7 ♂11, mean ± SD age: 22.8 ± 3.1	Standardized protocol of 4 familiarization repetitions shouldbe adopted for use in clinical practice and further research.	Reliability indicators between 5th, 6th, and 7th repetitions						
						ICC		SEM (%)		SDD (%)
				ANT		0.84		2.48		6.87
				ANTM		0.85		2.21		6.13
				ANTL		0.87		2.78		7.71
				M		0.86		2.67		7.40
				L		0.91		2.77		7.68
				P		0.92		2.79		7.73
				PM		0.86		2.94		8.15
				PL		0.92		2.62		7.11
SEBT	Robinson and Gribble (2008)	20 healthy individuals ♀10, mean ± SD age: 21.5 ± 3.3 ♂10, mean ± SD age: 23.2 ± 3.3	Normalized maximum excursion distance stabilized after approximately 4 familiarization repetitions	Not applicable						

ANT/ANTM/ANTL/M/L/P/PM/PL and CS, anterior/anteromedial/anterolateral/medial/lateral; posterior/posteromedial/posterolateral reach and composite score; RL/LL, right/left leg; ICC, interclass correlation coefficient; SEM, standard error of measurement; MDC, minimal detectable change; *p*-value, probability of type I error; SD, standard deviation.

### 3.6 Using a dedicated test kit during testing

The study conducted by Bulow et al. (2019) indicates that performing the test using a dedicated kit, compared to testing without equipment (using tape on the floor), results in statistically significantly lower scores for all directions and the composite score. Conversely, the results of the study by Jagger et al. (2020) show that performing the test using a dedicated kit, compared to testing without equipment, results in statistically significantly higher scores exclusively for the PL direction, with no significant differences for the other directions. Detailed data can be found in Table 9.

**TABLE 9:** Summary of study results on the impact of number of dedicated kit on test outcomes.

Test	Authors	Participants	Difference Magnitude/Stat. Significance/Effect size					Reliability indicators
mSEBT vs. YBT-LQ	Bulow et al. (2019)	25 ♀ healthy adolescents mean ± SD age: 14.0 ± 1.3			mSEBT	YBT-LQ	*p*-value (Cohen’s d)	Not applicable
			ANT (%LLL), mean ± SD	RL	94.9 ± 6.4	65.6 ± 5.1	<0.01* (5.1)	
				LL	96.1 ± 5.1	57.0 ± 4.5	<0.01* (8.1)	
			PM (%LLL), mean ± SD	RL	90.1 ± 10.8	100.3 ± 7.0	<0.01* (1.1)	
				LL	90.7 ± 9.2	101.0 ± 6.9	<0.01* (1.3)	
			PL (%LLL), mean ± SD	RL	83.2 ± 11.9	98.5 ± 7.8	<0.01* (1.5)	
				LL	83.8 ± 11.9	101.0 ± 7.9	<0.01* (1.7)	
			CS (%LLL), mean ± SD	RL	103.5 ± 10.9	102.1 ± 8.7	<0.01* (0.1)	
				LL	104.6 ± 10.7	103.6 ± 8.8	<0.01* (0.1)	
mSEBT vs. YBT-LQ	Jagger et al. (2020)	28 healthy adults (♀11, ♂53), mean ± SD age: 25.0 ± 2.2			mSEBT	YBT-LQ	*p*-value	Not applicable
			ANT (%LLL), mean	RL	65.4	64.8	≥0.05	
				LL	66.9	65.8	≥0.05	
			PM (%LLL), mean	RL	112.5	119.2	0.091	
				LL	112.5	119.6	0.061	
			PL (%LLL), mean	RL	103.9	113.0	0.021*	
				LL	102.5	112.3	0.018*	

%LLL, percentage of lower limb length, ANT/PM/PL and CS, anterior/posterolateral/posteromedial reach and composite score; RL/LL, right/left leg; *p*-value, probability of type I error, SD, standard deviation.

### 3.7 Heel lifting restriction

A database search did not reveal any studies on the impact of heel elevation on test outcomes.

## 4 Discussion

The aim of this systematic review was to compile studies verifying the impact of protocol variables on the outcomes of the “SEBT group,” including choice of calculation method, restrictions on arm movements, testing with footwear, warm-up procedures, the number of familiarization repetitions, the use of a dedicated test kit and restrictions on heel lifting. The study found that the choice of calculation method and arm movement restrictions have a significant impact on test results. Conversely, the influence of footwear, warm-up, and the use of a dedicated test kit remains unclear based on the available research. It also appears that the number of familiarization repetitions required to reach a plateau varies depending on the biological maturity level of the tested individual. A database search did not reveal any studies on the impact of heel elevation on test outcomes. As the first review to systematically compile the impact of these variables on the outcomes achieved, it offers a valuable source of information that can be useful from both a research and clinical practice perspective.

The results of studies by Sokulska et al. (2024) indicate that choosing a calculation method based on the maximum repetition, as opposed to a method based on the average of 3 repetitions, leads to higher test scores. Conversely, authors of studies analyzing the impact of the choice of calculation method on test reliability indicators have reached somewhat different conclusions. According to Shaffer et al. (2013) reliability indicators are more favorable for the method based on the average of 3 repetitions compared to the method based on the maximum repetition. In contrast, according to Kattilakoski et al. (2023) the calculation method does not affect reliability indicators. This study analyzed methods based on the first 3 repetitions, the best 3 repetitions, and the maximum repetitions. The discrepancies in the results may stem from differences in the test protocols. Shaffer et al. (2013) did not precede the test with a warm-up and performed 3 test repetitions following 6 familiarization repetitions. In contrast, Kattilakoski et al. (2023) preceded the test with a warm-up consisting of 5 min of walking followed by 5 min of jogging at a self-selected pace, and conducted 5 test repetitions preceded by 1 familiarization repetition. Based on the above data, it can be speculated that preceding the test with a warm-up reduces the dispersion of individual repetition results, which in turn makes the choice of calculation method less significant. The reduction in the variability of individual repetition results may be associated with the optimization of the postural control system due to warm-up, as observed by Paillard et al. (2018).

Research findings indicate that a test procedure allowing arm movements (compared to one with restrictions) enables achieving statistically significantly higher scores, at the cost of slightly reduced reliability (Hébert-Losier, 2017; Objero et al., 2019; Muehlbauer et al., 2022b; 2022a; Sogut et al., 2022). Higher scores obtained during testing with unrestricted arm movements can be attributed to at least 2 reasons. First, the arms act as a counterbalance, making it easier to maintain the vertical projection of the center of gravity within the base of support (Roos et al., 2008). Second, moving the mass away from the axis of rotation (outstretching the arms) increases the moment of inertia, which reduces angular accelerations, giving more time to perform corrective movements (Hill et al., 2019).

The study results do not allow for a definitive determination of the impact of wearing footwear during the test on its outcomes (Sogut et al., 2022; Park et al., 2023). Additionally, interpretation is hindered by the lack of mention in the cited studies regarding the standardization of the test protocol concerning heel elevation restrictions. On one hand, it can be speculated that wearing footwear during the test compensates for limitations in ankle dorsiflexion range of motion (by elevating the heel), which may be particularly important in testing procedures that prohibit heel elevation (Basnett et al., 2013; Olszewski et al., 2024). However, it is important to remember that differences in footwear design can be a confounding factor in the results. On the other hand, it can be assumed that performing the test barefoot allows for the precise acquisition of sensory information through the receptors located in the foot (Viseux, 2020). Additionally, it is important to note that moving in footwear is currently more natural for people than moving barefoot, making their postural control system operate in conditions closer to those encountered in daily life when tested with footwear.

The study results indicate an ambiguous impact of warm-up on “SEBT group” outcomes. The work of Bizzini et al. (2013) showed that after performing the FIFA 11+ warm-up, the composite score increased significantly. Imai et al. (2014) observed that a warm-up consisting of trunk stabilization exercises increased normalized reaches in the PM and PL directions as well as the composite score. This study did not observe changes in the effect of a warm-up consisting of conventional trunk exercises. Gogte et al. (2017) did not observe differences in the effects of passive, active, and mixed warm-ups on the composite score. Belkhiria-Turki et al. (2014) examining the impact of a warm-up consisting of a 5-min run combined with static or dynamic stretching of varying volumes, observed mainly unclear, trivial, or small effects. The discrepancy in research results is likely due to the diversity of warm-up protocols used by the authors. It can be assumed that each protocol prepared the body differently for the test task, which consequently led to differences in the results (van den Tillaar et al., 2019; McGowan et al.,2015). This observation indicates the need to develop a standardized warm-up protocol tailored to the needs of the test.

Most studies indicate that to stabilize the results in the “SEBT group,” it is necessary to precede the test with 4–6 familiarization repetitions in each direction for each leg. Based on the findings of Munro and Herrington (2010), as well as Robinson and Gribble (2008), it can be assumed that for testing adults, the number of familiarization repetitions should not be fewer than 4, while for adolescents, it should not be fewer than 6. In contrast, a study conducted by Onofrei et al. (2019) indicates that stable results can be achieved after just 1 familiarization repetition in the case of adult elite athletes who have experience performing the “SEBT group.” Based on the above observations, it can be assumed that an important criterion for selecting the number of familiarization repetitions is the level of biological development of the test subject. As indicated by Kiers et al. (2022), with the advancement of biological maturity, the efficiency of the postural control system increases, which, as the authors of this review suggest, may affect the effectiveness of adapting to the demands of the balance control test.

The comparison of research results conducted by Bulow et al. (2019) and Jagger et al. (2020) reveals ambiguity regarding the impact of using a dedicated test kit on the obtained results. Despite this ambiguity, the practical benefits advocate for testing with the use of a dedicated test kit.

### 4.1 Practical application

The practical application of the discussed findings can be distilled into specific recommendations aimed at maximizing performance and enhancing the reliability of the “SEBT group,” as follows:

#### 4.1.1 Choice of calculation method

The authors of the review recommend choosing a method of calculating results based on the average of 3 valid repetitions. Although this method may yield lower results compared to the maximum repetition method, it ensures results with better reliability indicators, which is crucial both from a research perspective and in clinical practice.

#### 4.1.2 Restrictions on arm movement

For testing healthy individuals, the authors of the review do not recommend implementing restrictions on arm movements, as the freedom to move arms makes the test more natural, albeit with a slight decrease in repeatability indices. However, restrictions on arm movements may be justified when testing individuals with specific conditions, such as anterior cruciate ligament reconstruction or chronic ankle instability, though this approach requires analysis in future studies.

#### 4.1.3 Wearing footwear during testing

The authors of the review do not recommend performing the test in shoes for two reasons. First, shoes introduce variability due to footwear design, mainly concerning the variability in the drop (the difference in height between the heel and toes), which can distort the results, especially in procedures that prohibit heel elevation. Second, performing the test barefoot may improves the quality of sensory information acquired by the foot and utilized by the postural control system. The authors believe that the most optimal solution for testing healthy individuals is performing the test barefoot with the possibility of heel elevation, although this approach requires analysis in future studies.

#### 4.1.4 Warm-up

Ambiguous evidence on the impact of warm-ups on test performance indicates the need to develop a standardized warm-up protocol tailored to the needs of the “SEBT group.” According to the authors of this review, the warm-up should be brief (up to 10 min), include simple exercises that do not require additional equipment, and specifically prepare the body for the test task.

#### 4.1.5 Number of familiarization repetitions

Conducting 4–6 familiarization repetitions before the test is recommended to enable participants to adapt adequately to the test requirements. Typically, 4 repetitions are sufficient for adults, while up to 6 repetitions may be necessary for adolescents due to their ongoing biological development. For elite athletes experienced in performing the test, fewer familiarization repetitions may be appropriate.

#### 4.1.6 Using a dedicated test kit during testing

The use of a specific test kit is recommended to standardize the testing process. This approach simplifies the procedure, promotes consistency, and helps in comparing results more effectively across various studies. While there are mixed results regarding its impact, the practical benefits of using a dedicated test kit, such as ease of use and standardization, are undeniable.

### 4.2 Limitations

The analysis focusing solely on the healthy population introduces certain limitations to our systematic review, considering the application of these tests in specific clinical entities. The diversity of purposes and clinical contexts in which these tests are used may justify deviations from the proposed protocols. Our review aimed to explore how specific protocol variations affect test outcomes, but it was not intended to establish rigid guidelines for conducting the test across every population. Therefore, while we strive to provide general guidelines on protocols, it’s important to remember the need for their adaptation to the specific clinical needs and characteristics of the populations being studied.

## 5 Conclusion

In conclusion, this review highlights the significant role of the choice of calculation method and arm movement restrictions on the outcomes of the “SEBT group.” It also notes the ambiguous impact of wearing footwear during testing, warm-up, and the use of a dedicated test kit on the results. Additionally, it appears that the number of familiarization repetitions required to reach a plateau varies depending on the biological development level of the tested individual. Future research should focus on developing a standardized warm-up protocol tailored to the needs of the “SEBT group,” and verifying the impact of heel lifting during testing on the obtained results.

## Data Availability

The original contributions presented in the study are included in the article. Further inquiries can be directed to the corresponding author.
